# Hypertension education and adherence in South Africa: a cost-effectiveness analysis of community health workers

**DOI:** 10.1186/1471-2458-14-240

**Published:** 2014-03-10

**Authors:** Thomas A Gaziano, Melanie Bertram, Stephen M Tollman, Karen J Hofman

**Affiliations:** 1 Division of Cardiovascular Medicine, Brigham & Women’s Hospital, 75 Francis Street, 02115 Boston, MA, USA; 2 Harvard Medical School, Boston, USA; 3 Harvard University School of Public Health, Boston, USA; 4 University of the Witwatersrand School of Public Health, Johannesburg, South Africa; 5 MRC/ Wits Rural Public Health and Health Transitions Research Unit (Agincourt), University of the Witwatersrand, Johannesburg, South Africa; 6 Centre for Global Health Research, Umeå University, Umeå, Sweden; 7 INDEPTH Network, Accra, Ghana

**Keywords:** Community health workers, Hypertension, Cost-effectiveness

## Abstract

**Background:**

To determine whether training community health workers (CHWs) about hypertension in order to improve adherence to medications is a cost-effective intervention among community members in South Africa.

**Methods:**

We used an established Markov model with age-varying probabilities of cardiovascular disease (CVD) events to assess the benefits and costs of using CHW home visits to increase hypertension adherence for individuals with hypertension and aged 25–74 in South Africa. Subjects considered for CHW intervention were those with a previous diagnosis of hypertension and on medications but who had not achieved control of their blood pressure. We report our results in incremental cost-effectiveness ratios (ICERs) in US dollars per disability-adjusted life-year (DALY) averted.

**Results:**

The annual cost of the CHW intervention is about $8 per patient. This would lead to over a 2% reduction in CVD events over a life-time and decrease DALY burden. Due to reductions in non-fatal CVD events, lifetime costs are only $6.56 per patient. The CHW intervention leads to an incremental cost-effectiveness ratio of $320/DALY averted. At an annual cost of $6.50 or if the blood pressure reduction is 5 mmHg or greater per patient the intervention is cost-saving.

**Conclusions:**

Additional training for CHWs on hypertension management could be a cost-effective strategy for CVD in South Africa and a very good purchase according to World Health Organization (WHO) standards. The intervention could also lead to reduced visits at the health centres freeing up more time for new patients or reducing the burden of an overworked staff at many facilities.

## Background

The burden of non-communicable diseases (NCDs) in low and middle income countries (LMIC) is very high and compounds the effects of the already high burden of infectious diseases [1]. Of the NCDs, hypertension is a major burden in general and in particular in South Africa with trends showing a growth of 20% for both men and women over the past decade [2]. Of further significance is that adherence to hypertension medication varies significantly from under 10% in the lowest socio-economic status (SES) quintile compared to 80% in the highest quintile [3]. Furthermore, HIV/AIDS can now be regarded as a treatable chronic illness, with the expectation that persons with HIV/AIDS will live longer and lead more active lives. This will increase their exposure to CVD risk including hypertension [4].

However, effective management of patients who are at high risk for NCDs in low-resource settings is challenging due to limited human and financial resources [5]. In response, the South African Department of Health has recently acknowledged the need for improved community based care for NCDs and is currently undergoing a major ‘re-engineering’ of its Primary Health Care (PHC) system [6]. The goal of the restructuring is to ensure the service capacity necessary to manage the ongoing infectious disease challenges in addition to the rising demands from increasing hypertension and other NCDs with limited numbers of doctors and nurses. The restructuring of the PHC will include the training of over 50,000 Community Health Workers (CHWs.) In addition to the traditional training for maternal and child health, HIV/AIDS, and tuberculosis, authorities have considered that training in Cardiovascular Disease management is necessary.

It is thus worthwhile to consider which lessons from prior initiatives with CHWs may be applicable to Cardiovascular Disease interventions. Currently, the utilization of CHWs in many low and middle income countries tends to focus on infectious disease management. Where CHWs have been used to manage NCDs, this has largely been for improving adherence and lifestyle choices, or screening for cancer. Finally, the WHO has also articulated the explicit recommendation for the existence of referral systems as part of managing care and for the appropriate training of health workers to use them [7]. However, to date, there is no evaluation of the cost-effectiveness of CHWs in aiding adherence to medications for cardiovascular disease (CVD).

We therefore propose to evaluate the cost-effectiveness of training CHWs to help with the adherence of stabilized hypertensive patients within the PHC system. If effective, this would serve two purposes. First, patients with the diagnosis of hypertension may benefit from improved control of their hypertension. Second, the staff at primary care facilities will be able to manage newly diagnosed conditions and not be overwhelmed, as patients are cared for in the community rather than depending on frequent PHC visits.

## Methods

### Strategies compared

We used a previously established Markov model with age-varying probabilities of CVD events to assess the benefits, risks, and costs of a program to increase hypertension adherence for individuals aged 25–74 in South Africa. In South Africa, under standard care 42% of the population is aware of their diagnosis, with only about 15% having their blood pressure controlled [8]. We evaluated the benefit of having CHWs visit patients with uncontrolled hypertension two times a year. This strategy was compared to usual care and control rates.

### Model description

In order to evaluate the full benefits and costs of the increased screening, we used Markov modeling with age-varying probabilities of cardiovascular disease events and mortality. The model has been described in detail elsewhere [9,10] but we describe it briefly here. The population distribution for adults between the ages of 25–74, by age and sex, was taken from the South African census data. The country-specific distributions of blood pressure and smoking rates, was taken from the South African demographic and health surveys of 1998 and 2003 updates [8,11]. Cholesterol values, by age and sex, were taken from the Global Burden of Disease project [12,13]. Diabetes distributions by age and sex were obtained from the Global Burden of disease with updates from the South African Medical Research Council study on the burden of diabetes [14].

Each year the cohort in each country faces a probability of death from a non-cardiac cause, developing coronary heart disease, having a stroke, or surviving free of CVD. The annual probability of non-cardiac death is based on life tables provided by the WHO for South Africa [15]. The risks for a first event if untreated—either coronary heart disease (CHD) or stroke—were based on separate Framingham risk functions for each event [16,17]. In the initial 35 days following an MI or stroke, individuals face various probabilities of surviving, having a repeat MI, dying from the MI, or having a bleeding complication or repeat stroke. After 35 days, survivors face separate probabilities of dying in the first year and in subsequent years based on age, and of having a debilitating stroke. Mortality and event probabilities for the first 35 days, the first year, and the following years for the group without secondary treatment were taken from the control cohort of the ISIS-2 [18] trial. Repeat events and subsequent CHD and stroke events were tracked for each individual, and affected mortality, quality-of-life, and costs, accordingly.

At the end of each year, the cohort is then redistributed to one of five health states. The five states are “disease-free” (no CVD event or death from other causes), “post-myocardial infarction (MI)”, “angina”, “post-cerebrovascular accident (CVA)”, and “dead”. For those in the disease-free state, the risk factors update with age, which updates annually. All analyses were performed using TreeAge Pro Suite 2009 by TreeAge Software Incorporated (Williamstown, Massachusetts). Table 1 lists the model input parameters for disease progression.

**Table 1:** Disease progression inputs used in the CVD micro-simulation model

**Parameter**	**Value**	**Source**
**From disease free state**		
Non-CVD death	Age- and sex-specific table	NCHS [19]
Stroke event	RF-based equation	Wolf [17]
CHD event	RF-based equation	Anderson [20]
% Cardiac arrest	Age- and sex-specific table	Weinstein [21]
% MI (males)	0.350	NHLBI [22], ARIC [23]
% MI (females)	0.200	NHLBI [22], ARIC [23]
% Angina	Formula	100% -% Cardiac arrest -% MI
**From cardiac arrest state**		
Acute (within 1 year) death	0.954	Nichol [24]
Chronic (post 1^st^-year) death	0.040	Assumption: same as MI
*From MI state*		
Acute death	Age- specific table	McGovern [25], Roger [26], Lee [27]
Acute CABG	0.082	Fang [28]
Acute PTCA	0.300	Fang [28]
% Procedure death	0.009	Dorros [29]
Acute 2^nd^ MI (after PTCA)	0.052	BARI [30]
Chronic (post 1^st^-year) death	0.040	Law [31]
>1 previous MI	0.100	Law [31]
Repeat MI	0.064	Jokhadar [32]
**From MI and CABG state**		
Acute post-CABG death	0.027	Peterson [33]
Acute 2^nd^ MI	0.051	BARI [30]
Chronic (post 1^st^-year) death	0.040	Assumption: same as MI
>1 previous MI	0.100	Assumption: same as MI
Repeat MI	0.039	Yusuf [34], Jokhadar [32]
**From angina state**		
Acute death	0.045	Capewell [35]
Acute cardiac arrest	0.006	Hsia [36]
Acute MI	0.035	Hemingway [37]
Acute CABG	0.200	Ford [38]
Acute PTCA	0.300	Ford [38]
Chronic (post 1^st^-year) death	0.030	Law [31]
Chronic (post 1^st^-year) MI	0.035	Hemingway [37]
**From angina and CABG state**		
Chronic (post 1^st^-year) death	0.018	Yusuf [34], Law [31]
Chronic (post 1^st^-year) MI	0.021	Yusuf [34], Hemingway [37]

### Effectiveness data

We rely on the only two studies using CHWs in the community for blood pressure control for the effect of the intervention- one in inner city Baltimore [39] and one in rural Taiwan [40]. Both observed a significant reduction in systolic blood pressure. The first, a randomized control trial of home visits for blood pressure management, observed a 3–6 mm reduction in blood pressure over 3.5 years, an effect which disappeared after home visits ceased. No difference was observed between those in the standard intervention group (one visit) and those in the more intensive intervention group (5 additional visits). In Taiwan, measurements taken in one city before and after a CHW intervention for blood pressure management found a 4-7 mm reduction in blood pressure at 3.5 years. We then applied the average blood pressure reduction from a meta-analysis of the trials [41] using these levels of treatment with an average of 22% reduction in coronary heart disease events and a 41% reduction in stroke per 10 mmHg reduction in systolic blood pressure. Table 2 describes the necessary inputs for this intervention.

**Table 2:** Intervention parameters applied to South Africa

**Parameter**	**Value**
Number of visits by CHW per hypertensive patient	2 per year
Reduction in blood pressure	4 mmHg (2–7 mmHg)
Number of households per CHW	
Average	260
Urban	300
Rural	225
Deep rural	150
Number of CHWs per Nurse coordinator	6
Number of training days/year	2

### Intervention description

We evaluated a simulation intervention where six CHWs, over a 2-day period, will be trained, to measure blood pressure using an automated blood pressure cuff, and about the etiology and prevention of hypertension and cardiovascular disease. Following training, each CHW would be given a list of hypertensive patients registered at a nearby clinic, with their home address. We estimate that each CHW could feasibly make 6 home visits per day based on a population density of approximately 2500 adults/5 km^2^, of which roughly 21% are hypertensive [8]. In rural areas we estimate up to four visits and in deep rural areas up to 3 visits a day due to the distances between households. Each CHW will be responsible for visiting each of their patients twice per year (approx. 245 working days) in order to monitor their blood pressure and treatment adherence, teach about healthy lifestyle choices and encourage follow-up visits with a doctor if necessary (which might require visiting the nearest district hospital). Both the CHW and the patient would record the patient’s blood pressure at each visit in order to monitor any changes. The CHW will report weekly to the Program Coordinator (usually a nurse supervisor) who will be responsible for supervising the work of up to 30 CHWs.

### Outcome measures and costs

Costs for the various inputs of the intervention including, CHW salaries, a nurse supervisor per five CHWs, and the training costs are listed in Table 3. The main goal of this hypertension adherence and education program is to prevent more serious sequelae of myocardial infarction, angina, and stroke. Potential cost savings of disease management of these acute CVD events and chronic care are listed in Table 4. Costs for treatment of myocardial infarction and stroke are based on an ingredients based approach where we calculate average number of hospital days per each event; physician, nurse, and hospital personnel costs; medication use, and laboratory testing. Input costs are taking from the WHO CHOICE (CHOosing Interventions that are Cost Effective) costs estimates. Costs were reported in $US for 2012.

**Table 3:** Intervention specific costs, South Africa

	**Unit cost in $US**
**Salary**	
Community Health Worker (CHW) annual salary	3750 (3187.5 – 4312.5)
Program coordinator annual salary	27933 (23743.05 – 32122.95)
**Training**	
Trainer daily salary	116.3875 (98.93 – 133.85)
CHW per diem	32.5 (27.63 – 37.38)
Trainer per diem	32.5 (27.63 – 37.38)
Room rental per day	31.25 (26.56 – 35.94)
Chairs	12 (10.20 – 13.80)
Desks	2.8625 (2.43 – 3.29)
Laptop computer	525 (446.25 – 603.75)
Projector	125 (106.25 – 143.75)
Projector screen	125 (106.25 – 143.75)
Notebook	0.125 (0.11 – 0.14)
Pencil	0.00875 (0.0074 – 0.010)
**Home visits and follow-up**	
Cell phone and minutes	15 (12.75 – 17.25)
Automated blood pressure cuff	87.5 (74.38 – 100.63)
Recording sheet for patient	0.04 (0.034 – 0.046)
Educational pamphlets	0.375 (0.32 – 0.43)

**Table 4:** Cost and utilities related to cardiovascular disease events in the model

**Parameter**	**Base-case value**	**Sensitivity analysis value(s)**
**Acute costs for disease states**		
MI	$1112	+/-15%
Stroke	$1564	+/-15%
**Chronic annual costs for secondary prevention**		
All CHD states	$300	+/-15%
Stroke	$900	+/-15%
**Blood pressure treatment costs**		
Annual treatment	$28.87-88.03	+/-15%
Annual lab costs	$6	+/-15%
**Disutilities for disease states**		
Acute MI	0.439	0.405-477
Angina	0.124	0.105-0.141
Acute stroke	0.92	+/-15%
Post stroke	0.266	0.228-0.295
**Disutilities for repeat event**		
Repeat MI event	-0.049	+/-15%
Repeat stroke event	-0.052	+/-15%

Outcomes in the analyses were measured in quality adjusted life-years gained and net health-care costs. Total life years and DALYs were accumulated for the population for both the standard of care currently in place and the CHW intervention. DALYs were obtained by use the weighted disease-state values from the Disability Weights of the WHO Global Burden of Disease project and are listed in Table 4.

#### Statistical analysis

Incremental cost-effectiveness ratios were calculated as the difference in costs between competing strategies divided by the increase in DALYs averted. To compare one strategy with the next more expensive alternative, we used incremental cost-effectiveness ratio (ICER), which is the difference in costs divided by the difference in DALYs. Sensitivity analyses on the effectiveness of the intervention relied on the upper and lower limits of the expected benefits from increased adherence. Specifically, we assessed the range of estimates on the number of visits depending on population density and the responsiveness to the reminders about medications and changes to the lifestyle advice received. Furthermore, we evaluated the absolute costs and reductions at increasing proportions of those responsive to the CHW program. Sensitivity analyses were conducted on the range of costs presented for CHW salary, the number of visits per year, the mortality from myocardial infarction, and costs of hospitalization. Given uncertainty around the predictive accuracy of the Framingham Risk Function outside of the United States [42] we tested whether the risk over or understanding ischemic heart disease in South Africa would have an impact on the results. Finally we conducted a probabilistic sensitivity analysis on the key variables of the model listed in the tables.

## Results

The cost of the intervention, including training, of CHWs visiting the household of a hypertensive patient two times per year is about $8 per patient per year. However, the costs are somewhat offset by reductions in 2% of non-fatal cardiovascular disease events over a life-time. As a result of these offsets, the net annual cost is less than $0.50 per person with only an additional life-time cost of $6.56 per patient. Table 5 lists the life time costs, effects, and incremental cost-effectiveness ratio for the intervention and the current standard. The CHW intervention leads to an incremental cost-effectiveness ratio of $320/DALY averted. This is well below a willingness to pay threshold of $2154 (Afro E region of WHO) or $10,000 (South Africa GDP per capita) for a “very good buy” set by the WHO’s CHOICE program.

**Table 5:** Cost, effects, and cost-effectiveness: CHW intervention against hypertension, South Africa

**Strategy**	**Cost**	**Incremental cost ($US)**	**DALY**	**Incremental DALY averted**	**C/E ($/DALY)**	**ICER**
Standard	2133.03		14.0508		151.81	
CHW	2139.59	6.56	14.0713	0.0205	152.05	320

DALY = Disability Adjusted Life Year; C/E = Cost-effectiveness; ICER = Incremental Cost-effectiveness ratio.

The cost-effectiveness of the intervention is somewhat sensitive to the cost of the intervention. One of the largest drivers of variation in costs was the number of households that could be visited by CHWs in regions of differing population density (Table 6). In urban areas the cost-effectiveness of the intervention was $17/DALY averted, compared with the ratio of $1529/DALY averted in deep rural areas where the number of visits per day were estimated to be half of that in an urban area. The cost-effectiveness of the intervention in widely spread ‘average’ rural areas lies between the two at about $772/DALY averted, which is about twice the national average.

**Table 6:** CHW vs standard intervention – by population density

**Population density**	**Incr C/E (ICER)**
Urban	17
Rural	772
Deep rural	1,529

ICER = Incremental cost-effectiveness ratio.

**Figure 1:**
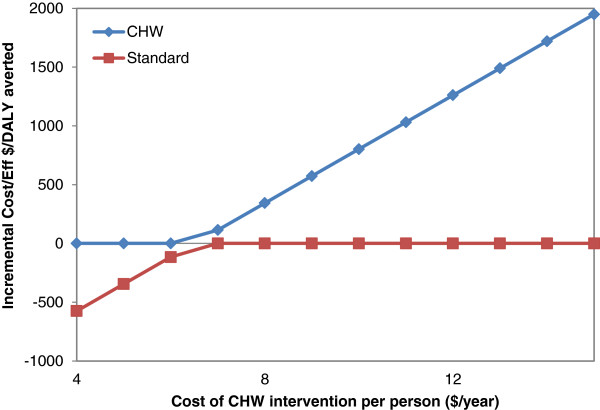
Effect of CHW intervention cost on incremental cost-effectiveness ratios (ICERs)*. Red line below zero results in a negative ICER which means that the CHW intervention is cost-saving.

**Figure 2:**
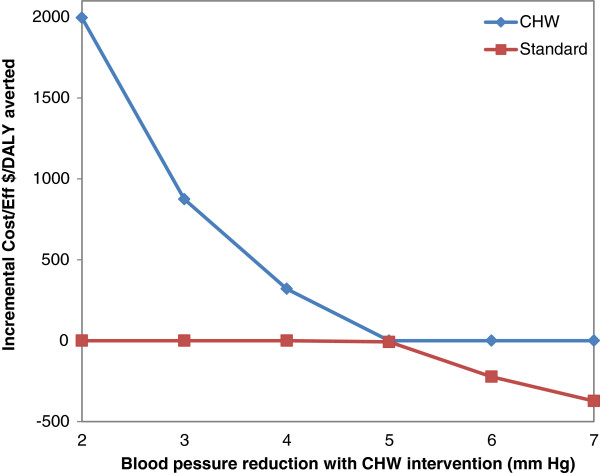
Effect of estimated BP reduction from intervention on incremental cost-effectiveness ratios (ICERs)*. *Red line below zero results in a negative ICER which means that the CHW intervention is cost-saving.

When we tested the sensitivity of the CHW intervention to the cost per patient, the intervention remained cost effective (Figure 1). Above our base case annual cost of $8 per patient the intervention remained cost-effective even with a near doubling of the cost at $15 per patient ($1900/DALY). Once the annual cost per patient was below $6.50, the CHW intervention became “cost-saving” (i.e. it both saved costs and increased life-expectancy). The results were also favorable across the full range of estimates of the benefit of the intervention on blood pressure reduction (Figure 2). At the levels below the base case assumption of 4 mmHg the intervention remained cost-effective down to a level of only 2 mmHg reduction where it was just under $2000/DALY averted. Once the blood pressure reduction was above 4.98 mmHg, the CHW intervention became cost-saving. Results were not sensitive to changes in the cost of hospitalizations or CHW salary; that is, the ICERs remained below $2000/DALY averted.

The results were not sensitive to a change in the mortality rate from myocardial infarction of 50% more or less than the baseline rate assumed in the model (ICERs for the CHW intervention compared to the standard of care ranged from ($305-$335/DALY averted. The results were also not sensitive to a cost of an MI ranging 50% above and below the base case value (range of ICERs $316-$326/DALY averted). When we assessed the predictive accuracy of the Framingham risk score, we found the results were somewhat sensitive to whether it over or underestimates risk in South Africans but all results remained well under the willingness to pay of $10,000 per DALY averted. If the risk score overestimates by up to 50% relatively an individual’s risk the ICER for the CHW intervention increases to $2991/DALY averted. If it underestimates the risk by 50%, the ICER for the CHW intervention becomes cost-saving, that is saves lives and costs less than the standard of care.

**Figure 3:**
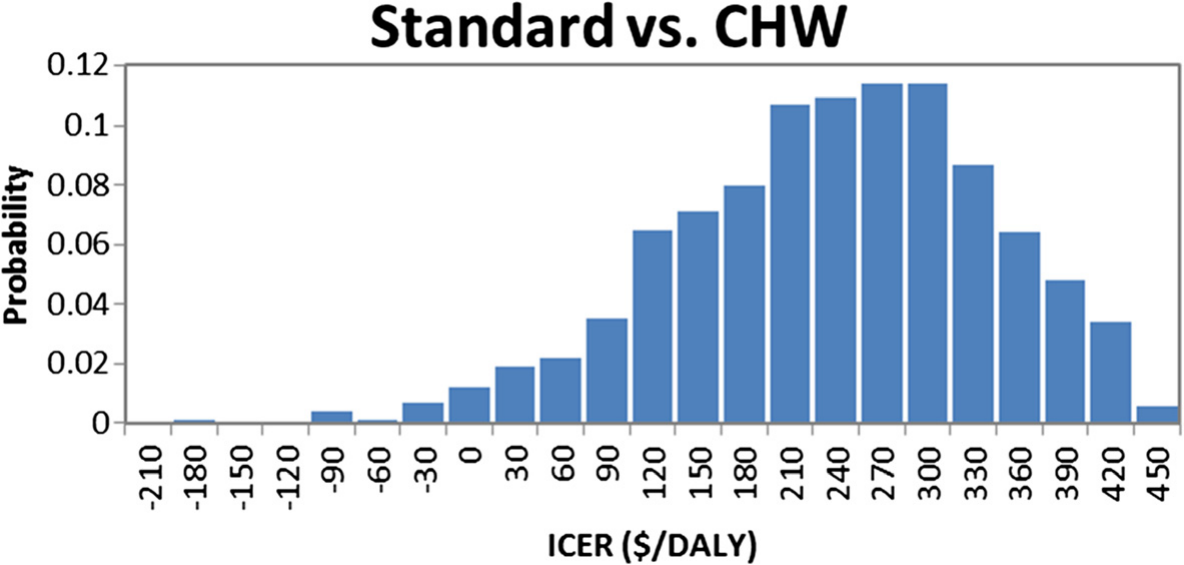
Probabilistic sensitivity analysis-distribution of incremental cost-effectiveness ratios*. *Negative ICER values reflect that the CHW intervention is cost-saving compared to the standard of care.

The results of the probabilistic sensitivity analysis (Figure 3) showed the results to be quite robust. The mean ICER was $223/DALY with 95% of the results remaining in an interval ranging from $0.40-$402/DALY averted. The maximum ICER comparing the CHW intervention to the standard of care was $441/DALY averted. The minimum values showed that the CHW intervention was cost-saving.

## Discussion

Our analyses have shown that an intervention of training CHWs to educate patients about the risk of hypertension and the benefits of life-style changes and adherence to medications would lead to cost-effective prevention of cardiovascular diseases at about $320/DALY averted. Even with a conservative estimate of a 2 mmHg reduction in systolic blood pressure, or almost a doubling of the cost of the intervention, the incremental cost-effectiveness ratio (ICER) remains below $2000/DALY averted. The intervention would lead to approximately 2% reduction in strokes and a 1% reduction in ischemic heart disease. Overall these estimates likely underestimate the benefit of the intervention as we did not include reductions in congestive heart failure and end-stage renal disease that are also affected by increased blood pressure. Another underestimation of the benefits includes the fact that when CHWs who encourage the adherence to one medication or lifestyle change they likely have an impact on other chronic conditions such as dyslipidemia and diabetes and thus could further reduce CVD and other chronic conditions. An emphasis on overall CVD risk has been shown to be more cost-effective [43] than just focusing on blood pressure and we ultimately suggest that CHW training on multiple CVD risk factors together may have a greater impact.

The WHO considers interventions to be cost-effective if they fall below three times gross-national income per capita. The ICERs we report in the main analysis and across the range of sensitivity analyses all fell below this threshold for South Africa as well as for most of the sub-Saharan African countries. Out main finding would be a “very good buy” according to WHO CHOICE criteria of less than one times gross national income (GNI) per capita in South Africa and neighboring countries. These values make this strategy a likely good option for many African and potentially other low and middle income countries.

Overall, there is a paucity of reported research on CHWs for CVD in developing countries; however, there is ample information about their use for infectious disease interventions in these settings. Several studies in developing countries have identified elements that are essential to the success of CHW interventions, such as community involvement in selecting CHWs, integration with existing health systems, adequate and ongoing training, and proper supervision [44,45]. Monetary payment for CHW services are useful and increasingly favored [46], though non-monetary incentives also help to reduce turnover [47]. These findings, which are similar to those from a review of CHWs in the United States [48], are likely to apply to CHW interventions for NCDs as well. Even a doubling of the CHW salary that we used would make this an attractive intervention.

Our study adds to the limited data available on the cost-effectiveness of task-shifting. Task-shifting from physicians to nurses in managing NCDs such as hypertension has been shown to be effective in several countries [49]. A review of the evidence regarding nurse-led interventions reveals that nurses are effective at the management of diabetes in primary care, outpatient, and community settings [50]; and in reducing hospitalizations, days spent in hospital, multiple readmissions, patient care, and cost-savings, even after factoring in the cost of the intervention [51]. One study in Pakistan [52] suggested that in addition to the education by community members, the general practitioners (GPs) taking care of the patients also had to have an adequate understanding of managing hypertension. In this study, GPs plus community education led to up to 10 mmHg reductions in blood pressure. While we believe the training in South Africa for hypertension management among doctors is adequate, nurse training in chronic diseases in many regions remains deficient. Still, the lack of human resources in LMICs overall, negatively impacts the ability of nurses to manage NCDs and the deployment of CHWs to offset this burden on nurses will be important. On average a hypertensive patient visits the primary health center 12 times a year to pick up medications and sees his or her nurse or physician 3–4 times per year. If the CHW visits can cut out 1–2 of these provider visits, then nurses would be freed up to either take on newly diagnosed patients with NCDs or better manage the already challenging case-load.

Another question is whether the CHWs can manage the workload. There are approximately 7.5 million South Africans with hypertension according to the 2003 WHO definition [53]. With only 42% aware of their diagnosis and only 15% controlled, there remain approximately 2.5 million who could benefit from improved hypertension management in the public sector that are already identified. Upwards of 50,000 CHWs are anticipated to be trained over the long term with the roll-out of primary health care re-engineering in South Africa. In this scenario, at capacity, each CHW takes responsibility for approximately 50 patients with hypertension. This is about one per 6 households that are expected to be managed by the CHW. The CHW could devote two of her expected 15–30 household visits per week (depending whether deep rural or urban) and comfortably accomplish this goal. Further, other care can be provided in this visit to increase economies of scale such as reminders about medications for other chronic conditions. In addition it appears that the intervention would even be cost-effective in remote areas where visits per CHW may be more challenging to achieve due to geography and population density.

The study has several limitations. First, the benefits of the intervention are based on studies outside of South Africa where, we used estimates from both a US study and one based in Taiwan. Other studies though have shown that task-shifting lead to improved treatment adherence for HIV medications and for mental health in South Africa and may be cost-effective [54-56]. A 20% increase in adherence may lead to a 4 mmHg blood pressure reduction [57,58]. Additional gains with lifestyle advice and visit reminders could lead to further reductions. A second limitation is the use of WHO CHOICE results for event cost estimates. Until such local South African data can be procured through additional studies, we are reliant on WHO provided estimates. However, our cost estimates are quite conservative and any increase in the cost of CVD events would lead to more favorable cost –effectiveness ratios. Furthermore, the ICERs were quite robust even when a doubling of the cost of the intervention was assessed. A third limitation is our assumption that the benefit would be the same for all patients. However, there may be differences in responsiveness to the intervention based on geographic location, level of prior experience with CHWs in certain areas of South Africa (primarily urban), or income of the patients. Further work is needed to assess if these differences exist. One of the key conclusions is that this CHW intervention addressing both adherence and identification has the potential to be especially important for the lowest quintiles of the population, many of whom live in the rural and deep rural areas of South Africa.

## Conclusion

This study found that CHWs could potentially have a significant impact on chronic conditions in South Africa and other middle-income countries leading to improved blood pressure control and reduced strokes and myocardial infarctions. The demands on CHWs in South Africa and elsewhere are going to continue to grow as the health transition unfolds and CVD burden grows while health care professional numbers continue to be less than adequate for the need. Questions regarding how best CHWs can contribute to the overall care and education of the public will remain. However, it appears that training CHWs to improve community knowledge and individual adherence to medications for hypertension (and potentially other chronic non-communicable conditions) may be one valuable use of scarce human resources for one of the leading causes of death and disability in South African adults.

## Abbreviations

CHW: Community health worker; CVD: Cardiovascular disease; ICER: Incremental cost-effectiveness ratio; DALY: Disability adjusted life-year; WHO: World Health Organization; LMIC: Low- and middle-income countries; HTN: Hypertension; SES: Socio-economic status; NCD: Non-communicable disease; PHC: Primary health care; CHD: Coronary heart disease; MI: Myocardial infarction; CVA: Cerebrovascular accident; CHOICE: CHOosing interventions that are cost effective; GDP: Gross domestic product; GNI: Gross national income.

## Competing interests

There are no conflicts of interest.

## Authors’ contributions

All four authors were involved in the design of the study. TAG designed the Markov model and conducted the analyses using the model. MB and KJH contributed to the model inputs. TAG wrote the initial draft of the paper and all other authors contributed to the review of the paper. All authors read and approved the final manuscript.

## Authors’ information

Stephen M Tollman: http://www.indepth-network.org

## Pre-publication history

The pre-publication history for this paper can be accessed here:

http://www.biomedcentral.com/1471-2458/14/240/prepub
